# Editorial: Neonatal host immune responses to pulmonary infections

**DOI:** 10.3389/fimmu.2023.1219844

**Published:** 2023-05-24

**Authors:** Kerry M. Empey, Elizabeth D. Fixman, Stephania Cormier, Jay K. Kolls, Giovanni Piedimonte

**Affiliations:** ^1^ School of Pharmacy, University of Pittsburgh, Pittsburgh, PA, United States; ^2^ Department of Immunology, School of Medicine, University of Pittsburgh, Pittsburgh, PA, United States; ^3^ Meakins-Christie Laboratories, Research Institute, McGill University Health Center, Montréal, QC, Canada; ^4^ Pennington Biomedical Researcher Center and Department of Biological Sciences, Louisiana State University, Baton Rouge, LA, United States; ^5^ Tulane Center for Translational Research in Infection and Inflammation, School of Medicine, Tulane University, New Orleans, LA, United States; ^6^ Department of Pediatrics, Biochemistry and Molecular Biology, Tulane University, New Orleans, LA, ZUnited States

**Keywords:** infant immunity, lung infection, innate immunity, adaptive immunity, immune development and maturation

Infants are more susceptible to severe pulmonary infections compared to older children and adults. Historically, this has been attributed to an immature host immunity in the early months after birth. However, recent evidence describes a decidedly more complex and strategic infant immune response that is heavily influenced by a variety of factors. Understanding these factors and how they influence acute and subsequent responses to pulmonary pathogens along with strategies to mitigate long-term sequelae are the focus of this Research Topic, titled *Neonatal Host Immune Response to Pulmonary Infections.* In total, Frontiers in Immunology published 8 articles prepared by 56 authors from 5 countries, covering topics related to 1) factors that influence adaptive and innate immune responses to early life infection, 2) mechanisms of viral associated asthma, and 3) preventative/treatment strategies.

In the first line of research, Eddens et al. references staggering estimates of over 100 million lower respiratory tract infections each year in children under 5 years of age, which account for up to 900,000 deaths annually. Authors cite factors contributing to infection risk that include reduced innate immune cell function and a tolerogenic adaptive immune response with reduced memory T cell formation in infants compared to adults. Authors describe a Th2 skewed immune response to three leading causes of respiratory morbidity and mortality in young children – respiratory syncytial virus (RSV), human metapneumovirus (HMPV), and rhinovirus (RV) – that is largely triggered by epithelial cell-derived alarmins that contribute to long-term pathologic changes in some models. In a separate, but complementary review, Pieren et al. describe a “phasic shift” in the adaptive immune response to early life pulmonary infections. Present at birth, authors define Phase I as a rapid expansion of neonatal T cells that express TLRs for non-specific pathogen recognition, but which comes at the expense of poor long-term memory. Phase 2 marks a transition period to Phase 3, whereby memory cells become the main pool of cells that confer protection against pathogens and allow for long-lasting vaccine-mediated protection. The authors suggest that timing of infection is critical and phase-specific tolerogenic features may be used as biomarkers to personalize vaccination strategies.

Maternal infections during pregnancy constitute another factor that contributes to immune development and risk of lung dysfunction in children. The review by Manti et al. describes immediate and long-term consequences of maternal infection. Specifically, influenza infection during pregnancy is reported to increase the risk of prematurity, respiratory and neurological illness, and congenital anomalies. RSV exposure *in utero* is reported to cause selective immune deficits, airway remodeling, and abnormal airway smooth muscle contractility, which the authors postulate may predispose the child to subsequent airway hyperreactivity. Lastly, authors cite a high prevalence of premature births in pregnant women infected with SARS-CoV-2. Together these manuscripts highlight critical functional changes that occur during infant or maternal infection that alter subsequent immune responses.

A second line of research examines mechanistic links between pulmonary viral infections and subsequent development of asthma and wheezing. Zhang et al. show that adoptive transfer of eosinophils harvested from mice infected with RSV as neonates and subsequently sensitized and challenged with ovalbumin promote pulmonary pathology in naïve mice in response to ovalbumin challenge. CD4^+^ T cells increased in these mice, leading the authors to conclude that these eosinophils have the ability to present antigen to enhance T cell-dependent lung inflammation. In a two-hit murine model of RV infection, Han et al. used LysM^Cre^ IL-4Rα KO mice lacking M2a macrophages to show that ILC2-mediated type 2 cytokine production and mucus metaplasia are reduced in the absence of M2a macrophages following repeat RV exposure. Authors show that compared to wild-type infant mice infected with RV, lungs of mice lacking M2a had reduced IL-33, IL-25, and TSLP following heterologous RV infection and postulated that M2a macrophages and ILC2s work together to promote the development of Type 2 inflammation following RV infection. In a human cohort of children ages 2-3 years, Chirkova et al. showed that RSV infection in infancy alters subsequent immune responses to RSV. Children infected with RSV during infancy had significantly lower memory T cell responses to *in vitro* stimulation with RSV. This dampened T cell response occurred irrespective of the severity of RSV infection in infancy. These studies support the premise that early life infection alters immune development and predisposes to enhanced morbidity with subsequent respiratory infections.

A third line of research involved strategies to prevent or treat severe pulmonary infections in infancy and long-term sequelae. In a rhesus macaque model of BCG vaccination, Sarfas et al. demonstrated that immunized infant macaques generate a functional immune response to the vaccine, but responses were significantly lower than those observed in adults. Moreover, infant immune responses favor the activation and attraction of inflammatory macrophages and monocytes. The authors postulate that enhanced innate immune responses may contribute to BCG’s observed protection against non-mycobacterial organisms. Lastly, in a murine model of neonatal bronchopulmonary dysplasia (BPD) with hyperoxia, Cui et al. showed increases in lung F-actin-mediated inflammatory CD103+ dendritic cells and airway hyperreactivity following RV infection. Using *in vivo* and *ex vivo* approaches, authors showed that gelsolin, an F-actin severing protein, decreased F-actin levels in hyperoxic bronchoalveolar lavage fluid, blocked hyperoxia-induced CD103+ DC expansion and inflammation, and attenuated hyperoxia-induced hypoalveolarization that is commonly observed in children with BPD. The authors conclude that gelsolin may provide a promising option to treat RV-induced BPD exacerbations and prevent associated chronic lung disease.

Together, this Research Topic supports the paradigm shift in our thinking of infant immunity as being “immature” to one that recognizes pathogens with strong innate-like effector functions that are kept in check by a tolerogenic adaptive immune system, but which may also contribute to subsequent development of wheezing, asthma, and other chronic lung diseases. Strategies for the treatment and prevention of severe pulmonary infection and associated long-term sequelae depend on our understanding of infant host immune responses. This Research Topic highlights the need for continued studies to better elucidate age-dependent differences in host immune response to pulmonary pathogens and how early life infections alter immune development.

## Author contributions

KE: conceptualization, wrote the original draft; EF: review and editing; SC; review and editing; JK: review and editing; GP: review and editing. All authors contributed to the article and approved the submitted version.

